# Knowledge and perceptions of synthetic cannabinoids among university students in Jordan

**DOI:** 10.1371/journal.pone.0253632

**Published:** 2021-06-24

**Authors:** Mahmoud M. AbuAlSamen, Tamam El-Elimat, Basima A. Almomani, Nour A. Al-Sawalha

**Affiliations:** Faculty of Pharmacy, Jordan University of Science and Technology, Irbid, Jordan; Imam Abdulrahman Bin Faisal University, SAUDI ARABIA

## Abstract

The emergence of blends of synthetic cannabinoids (SC) is an alarming public health concern in Jordan and worldwide. This study aimed to assess the knowledge and perceptions of university students of SC. A cross-sectional study employing a self-administered questionnaire was used to collect data from 1,789 university students in Jordan. The questionnaire measured the knowledge and perceptions of students of SC. Bivariate and multivariate logistic regression were used to investigate the potential predictors of good knowledge of SC. Perceptions of students regarding SC use, prevalence and availability were investigated using principal component analysis. Self-reported familiarity with SC was high (92.5%), while good knowledge was only demonstrated by (33.6%) of students. Predictors of good knowledge included being a smoker (*aOR* = 1.369, 95% Cl = 11.041–1.871, p = .026), an alcohol user (*aOR* = 2.134, 95% CI = 1.362–3.346, p = .001), being informed by traditional media (*aOR* = 1.367, 95% CI = 11.113–1.679, p = .003), social media (*aOR* = 1.241, 95% CI = 1.161–1.403, p = .021) and self-familiarity with SC (*aOR* = 2.499, 95% CI = 1.518–4.114, p < .0001). Students perceived SC use to be prevalent and ethically unacceptable, for religious, social, and legal reasons. There were significant differences in the ethical perceptions against the use of SC detected by gender (p < .0001), smoking (p < .0001) or alcohol use (p = .001), and being informed by both traditional media (p-.001) and social media (p = .001), but there were no differences by the level of knowledge (p = .057). Those of good knowledge and those of low knowledge did not differ on their ethical perceptions of using SC. This study showed that there was a low level of knowledge regarding SC among university students in Jordan, which may play a role in the use of SC in the country. Herein, many opportunities exist for public health education to raise awareness against SC use.

## Introduction

Synthetic cannabinoids (SC) constitute a large and growing family of recreational drugs [1]. Basically, they interact with the cannabinoid receptors 1 and 2 (CB1 and CB2) producing cannabimimetic effects that are similar to the naturally occurring cannabinoid [Δ^9^-tetrahydrocannabinol (Δ^9^-THC)], the key psychoactive constituent of cannabis [2, 3]. However, SC act as full agonists with high-potency and efficacy in comparison with the partial agonist Δ^9^-THC [4]. SC were developed originally as research tools to study the endocannabinoid system as potential therapeutic agents [1, 5]. SC are usually sprayed on herbal products for consumption. They emerged in the market as designer drugs in herbal smoking mixtures under several names including ‘JOKER’, ‘Spice’ and ‘K2’ [1, 6]. These compounds were captured by drug dealers and are being manufactured after structural modifications in clandestine laboratories for illicit consumption without a guarantee of quality, purity, and safety [7]. SC were brought into public attention as hazardous substances in 2008 [8, 9]. Consumption of SC has been associated with severe toxic effects such as acute kidney injuries, ischemic strokes, myocardial infarctions, and seizures [1, 10].

The systematic study of SC is hurdled by the continuous emergence of newly developed drugs. Once a SC has been identified in illicit products, novel SC are introduced into the market to evade legal consequences [1]. Hence, since 2010, SC were introduced into the market as waves. Between 2010 and 2012, the majority of SC reported in the US were JWH compounds, mostly JWH-018 and AM-2201 [11]. Between 2013 and 2015, XLR11, AB-FUBINACA, and AB-CHMINACA were the most frequently identified SC [12]. In Jordan, SC appeared under the name of ‘JOKER’. In this regard, the most commonly identified in the last four months of the year 2016 were AB-FUBINACA, XLR11, and AB-CHMINACA, with 5,409, 3,132, and 2,977 reports, respectively [13]. These drugs are now legally banned since the new Drug Abuse and Psychoactive Substances Act was enacted in 2016 [14].

Between 2014 and 2016, there was a SC crisis characterized by an outbreak of positive intake cases and SC abuse related crimes in Jordan. Statistically, in the last four months of the year 2016, a total of 11,518 positive cases of SC intake were reported in Jordan [13], making it the top-consumed illicit drug in Jordan [15]. Moreover, statistics from 2015 and 2016 showed that the number of SC addicts in Jordan who sought treatment at the Drug Rehabilitation Center in Arjan (affiliated with the Drug Enforcement Administration) was 559 and 995 cases, respectively [16]. Noticeably, most data of SC use in Jordan come only from clinical populations collected by local authorities and rehabilitation centers [13, 15, 16].

With this continual emergence of new SC, and the scarcity of information regarding their pharmacology, toxicology, and pharmacokinetics, as well as their detection, these compounds pose a great challenge to the law enforcement agencies and to the clinical and forensic toxicologists. There is evidence that SC use is particularly prevalent among the young [17]. Hence, this study examined the knowledge and perceptions of university students in Jordan about SC.

## Materials and methods

### Study design

A cross-sectional questionnaire-based study was conducted at nine universities in Jordan; five public and four private universities. Jordan has 10 public and 17 private universities. The study took place at universities that were located in six different governorates (Irbid, Jerash, Amman, Al-Mafraq, Az-Zarqa’ and Al-Karak) encompassing the northern, central, and southern areas of Jordan. A self-administered questionnaire was distributed to undergraduate students from different fields in the period between November 2017 and March 2018. The obtained sample was representative of young student population in Jordan.

### Participant recruitment and sampling

Prior to data collection, each university administration was approached for approval on data collection. From each university, we sampled students from courses which were representative of total university students. The courses that were chosen for participant recruitment were offered at all Jordanian universities as they were mandated by the Ministry of Higher Education and Scientific Research in Jordan. Moreover, enrolment in each of these courses was mandatory for all university students across all study plans and undergraduate programs, attendance was mandatory and students from all years attended these courses. Trained research assistants gave a brief description of the study aims to the students, and all the students inside class halls were invited. Students who expressed their interest to participate were further interviewed by the research assistants for eligibility, including: (1) being enrolled in an undergraduate program, (2) willingness to participate. Each participant read the consent form on the questionnaire front page, and students who consented, completed the questionnaire, and returned it. All participants recruitment, data collection, and the consent process were solely handled by the research assistants without any interference from the university officers or course instructors. The study was approved by the Institutional Review Board at Jordan University of Science and Technology (protocol number 281/2017, approval reference: 8/109/2017).

### Sample size

Based on a precision of 0.05 and 95% confidence interval, the sample size was estimated to be 385. To account for clustering, the estimated sample size was multiplied by design effect (DE) where DE = 1+ (m-1) × ICC, with m denoting the number of participants sampled per cluster and ICC denoting the intra-cluster correlation coefficient. ICC relates the variance within clusters with the variance between clusters and in the current study it was estimated to be 0.02 based on analysis from the pilot study. Based on 4 hours data collection window per day, we aimed to sample 150 students from each university within the allocated visit days. Each university was considered a cluster and the final sample size was calculated as 1533 participants. To account for non-response and incomplete responses, the number of students sampled at each university was increased.

### Questionnaire development

The questionnaire was developed in English language based on literature review and through discussion within the research team [18]. The questionnaire was comprised of closed-ended questions and the approximate time to complete the questionnaire was 10 min. The questionnaire consisted of three parts; (1) demographics, (2) self- familiarity and knowledge of SC, (3) perceptions of SC. The questionnaire was piloted in a small number of participants before conducting the study (n = 56) to assess the clarity of expression and wording of questionnaire items. The piloting data was not included in the final analysis. The final form of the questionnaire in Arabic (S1 Table) and English (S2 Table) are available as part of the Supporting Information.

Self-familiarity was defined as whether the participant has ever heard about SC. Knowledge was measured using 10 items with three answer options (true, false, I do not know). Good internal consistency of the knowledge section was shown by both Cronbach’s alpha = 0.78 and MacDonald’s Omega = 0.81.

The perceptions section was composed of two sections consisting of 8 items that measured students’ perceptions on a 5-point Likert scale (1: strongly disagree, 2: disagree, 3: neutral, 4: agree, 5: strongly agree). The first section was related to the ethical perceptions of SC use. The second section was related to the perception of SC prevalence and availability. Internal consistency of the perceptions section was measured by Cronbach’s alpha and was found to be 0.67.

### Statistical analysis

Statistical analysis was done in the Statistical Package for Social Sciences (SPSS) for Mac V.23 and JASP Statistical Package Version 0.9.2.0. Data were checked for normality using Kolmogorov-Smirnov test. Continuous variables were expressed as mean and standard deviation, while categorical variables were expressed as numbers and percentages. Internal consistency for the knowledge items was evaluated by both Cronbach’s alpha and MacDonald’s Omega. The answers to the 10 different questions regarding the knowledge of each participant were calculated by applying a cut-off point for total scores. A participant had good knowledge if the sum of the scores was ≥6 (out of 10) or had low knowledge if the sum of the scores was < 6 (out of 10).

The level of knowledge was taken as a dichotomous dependent variable and all respondents who gave the wrong answer or stated “do not know” were coded as “false”. In addition, for the purpose of analysis, we restricted response options for the perception section. For example, we combined both "strongly disagree" and "disagree" as one category and both "strongly agree" and "agree" as one category.

To investigate the association between students’ knowledge of SC and demographic factors, a univariate logistic regression analysis was conducted and variables that were significant at p < .20 were included in the multivariate analysis using the enter method. Univariate analysis is available as part of the (S4 Table). All the variables were tested for multi-collinearity, and no multi-collinearity was detected. Statistical significance was set at p <0.05.

Since perception is a latent construct, items in perceptions section were entered to Principal Component Analysis (PCA) in an attempt to extract meaningful factors that define the perceptions of students regarding SC. This statistical technique is recommended for latent variables measured using Likert-scale in public health research [19]. To check for factorability, both the Kaiser-Meyer-Olkin measure of sampling adequacy and Bartlett’s test of sphericity were checked. Factors were extracted at eigenvalues higher than 1.00 and by inspection of the breaking point in the scree plot. A parallel analysis in JASP software was conducted to confirm the solution. The full factorial structure of students’ perceptions of SC is provided as (S3 Table). Factors were scored using the regression method and used as dependent variables in further analysis. *t*-test or one-way analysis of variance (ANOVA) followed by Bonferroni *post hoc* test were used as appropriate.

## Results

### General characteristics of participants

A total of 2,050 questionnaires were distributed to students and 1,824 students filled the questionnaire at a response rate of 88.9%. Screening for completed questionnaires resulted in only 1,789 responses to be included in analysis. Most participants were females (60.1%), from public universities (63.8%) and were aged 20–24 years (65.7%). About 28% of the participants were enrolled in health-related fields and around half of participants (51.9%) were living in central Jordan. The majority of participants were non-smokers (57.1%) and a few of them consumed alcohol (5.5%). The general characteristics of the sampled students are shown in Table 1.

**Table 1:** Demographic characteristics of students participating in the study (n = 1,789).

Variable	N (%)
**Gender**	
Male	714 (39.9)
Female	1075 (60.1)
**Age**	
<20 years	498 (27.8)
20–24 years	1174 (65.7)
>24 years	117 (6.5)
**University**	
Public	1141 (63.8)
Private	648 (36.2)
**Religion**	
Islam	1685 (94.2)
Christianity	61 (3.4)
Other	43 (2.4)
**Smoking and waterpipe use (lifetime)**	
Non-smoker	1022 (57.1)
Smoker	665 (37.2)
Ex-smoker	102 (5.7)
**Alcohol consumption**	
Yes	99 (5.5)
No	1690 (94.5)
**Field of study**	
Medical	497 (27.8)
Engineering	426 (23.8)
Law, Humanities and Educational Sciences	250 (14.0)
Technology	125 (7.0)
Administrative	201 (11.2)
Arts and others	290 (16.2)
**Academic level**	
1^st^ year	426 (23.8)
2^nd^ year	382 (21.4)
3^rd^ year	412 (23.0)
4^th^ year	375 (21.0)
5^th^ and 6^th^ years	194 (10.8)
**Residency**	
Northern Jordan	464 (25.9)
Central Jordan	928 (51.9)
Southern Jordan	397 (22.2)

### Participants’ level of knowledge

The majority of participants self-reported high familiarity with SC (N = 1655, 92.5%). A tenth (11.4%, n = 204) of participants knew someone who was consuming SC. Questions in the knowledge section received a varying pattern of responses. One-third of the participants (33.6%, n = 601) were knowledgeable about SC. An overview of the knowledge statements is presented in Table 2. Statement # 7 (Synthetic cannabinoids may cause behavioral changes) and 8 (Synthetic cannabinoids may cause health problems) had a higher number of correct responses compared to other statements. On the other hand, statements #3 "Insecticides are used in manufacturing SC products" and #6 (Synthetic cannabinoids may cause death) were the ones with the lowest rate of correct responses.

**Table 2:** Participants’ responses to knowledge statements.

Statement	Correct answer N (%)	Incorrect answer N (%)
1. Synthetic cannabinoids are herbal substances	657 (36.7)	1132 (63.3)
2. Synthetic cannabinoids are drugs of pharmacological effect	1284 (71.8)	505 (28.2)
3. Insecticides are used in manufacturing synthetic cannabinoids products	81 (4.5)	1708 (95.5)
4. Synthetic cannabinoids are more potent than cannabis/ hashish	1295 (72.4)	494 (27.6)
5. Synthetic cannabinoids products are cheap	753 (42.1)	1036 (57.9)
6. Synthetic cannabinoids may cause death	91 (5.1)	1698 (94.9)
7. Synthetic cannabinoids may cause behavioural changes	1641 (91.7)	148 (8.3)
8. Synthetic cannabinoids may cause health problems	1592 (89.0)	197 (11.0)
9. Synthetic cannabinoids are hard to detect in blood or urine tests	221 (12.4)	1568 (87.6)
10. Marketed products containing synthetic cannabinoids are constantly changing	715 (40.0)	1074 (60.0)

Results from the multivariate logistic regression model (Table 3) showed that smokers had a higher level of knowledge compared to non-smokers (*aOR* = 1.369, 95% Cl = 11.041–1.871, p = .026). Alcohol users were also 2 times more likely to have good knowledge of SC than non-users (*aOR* = 2.134, 95% CI = 1.362–3.346, p = .001). Participants who were informed by traditional media (TV, radio, newspapers) (*aOR* = 1.367, 95% CI = 11.113–1.679, p = .003) and social media had a higher level of knowledge (*aOR* = 1.241, 95% CI = 1.161–1.403, p = .021) compared to those who were not. Moreover, students who were familiar with SC were 2.5 times more likely to have higher level of knowledge compared to who were no (*aOR* = 2.499, 95% CI = 1.518–4.114, p < .0001). Age, gender, residency, field and years of study were all not significantly associated with good knowledge of SC.

**Table 3:** Adjusted odds ratios and 95% confidence interval for predictors of good knowledge on synthetic cannabinoids.

Variable	aOR	95% CI	P-value
**Smoking/ waterpipe (lifetime)**			
Non-smoker	Referent		
Current smoker	1.396	1.041–1.871	.026
Ex-smoker	1.568	0.754–3.263	.20
**Self-reported familiarity with synthetic cannabinoids**			
No	Referent		
Yes	2.499	1.518–4.114	< .0001
**Alcohol consumption**			
No	Referent		
Yes	2.134	1.362–3.346	.001
**Informed by traditional media**			
No	Referent		
Yes	1.367	1.113–1.679	.003
**Informed by social media**			
No			
Yes	1.241	1.161–1.403	.021

Adjusted for age and gender

### Students’ perceptions of synthetic cannabinoids use

Items measuring students’ perceptions of SC use, prevalence and availability are shown in Table 4. Normalized scores from PCA are shown in Table 5.

**Table 4:** Students’ perceptions of synthetic cannabinoids use.

**Ethical Perceptions of synthetic cannabinoids use**	**Agree N (%)**	**Neutral N (%)**	**Disagree N (%)**
Taking synthetic cannabinoids is considered a behaviour banned by religion.	1557 (87.0)	81 (4.5)	151 (8.4)
Taking synthetic cannabinoids is considered a behaviour rejected by social norms.	1658 (92.7)	87 (4.9)	44 (2.5)
Taking synthetic cannabinoids is considered a behaviour banned by law	1666 (93.1)	82 (4.6)	41 (2.3)
Taking synthetic cannabinoids is considered a freedom of choice for the individual.	219 (12.2)	221 (12.4)	1349 (75.4)
**Perceptions of synthetic cannabinoids use prevalence and availability’**	**Agree**	**Neutral**	**Disagree**
It is easy to obtain synthetic cannabinoids from the local market.	888 (49.6)	567 (31.7)	334 (18.7)
The media reports on synthetic cannabinoids increase people’s curiosity to attempt taking synthetic cannabinoids.	804 (44.9)	547 (30.6)	438 (24.5)
The act of taking synthetic cannabinoids is widely spread among university students.	820 (45.8)	673 (37.6)	296 (16.5)
Social media can be utilized to spread awareness on the risks of taking synthetic cannabinoids.	1606 (89.8)	117 (6.5)	66 (3.7)

**Table 5:** Perceptions of synthetic cannabinoids use by Jordanian university students.

Variable	Ethical Perceptions of SC Use		Perception of SC Prevalence and Availability	
	Mean ± SEM	*p*-value*	Mean ± SEM	*p*-value*
**Gender**		< .0001		.028
Male	-0.21 ± 0.05		-0.07 ± 0.04	
Female	0.07 ± 0.02		0.03 ± 0.02	
**Smoking**		< .0001		.024
Non-smoker	0.05 ± 0.01		-0.05 ± 0.03	
Smoker	-0.30 ± 0.06		0.11 ± 0.06	
Ex-smoker	-0.40 ± 0.24		0.20 ± 0.21	
**Informed by traditional media**		.001		.225
Yes	0.06 ± 0.02		-0.02 ± 0.02	
No	-0.07 ± 0.03		0.02 ± 0.03	
**Informed by social media**		.001		.002
Yes	0.05 ± 0.02		0.05 ± 0.02	
No	-0.07 ± 0.03		-0.06 ± 0.03	
**Level of knowledge**		.057		.001
Knowledgeable	0.09 ± 0.03		0.34 ± 0.02	
Not knowledgeable	-0.03± 0.02		-0.16 ± 0.02	

*p-value is two tailed and was computed either by t-test or one-way ANOVA as appropriate. SEM: The standard error of the mean.

Scores showed how far the different groups perform on a latent construct and a higher mean indicated a higher score of that perception. Data analysis showed that ethical justifications against the SC were significantly different by gender (*p* < .001), where females scored higher than males. Additionally, omnibus analysis of variance showed that smoking had an effect on these perceptions where smokers scored the least compared to non-smokers and ex-smokers (*p* < .0001). Furthermore, those who had been exposed to information regarding SC by traditional and social media showed significantly higher scores than those who had not been informed by both traditional media (*p* = .001) and social media platforms (*p* = .001). However, abstinence from using SC for ethical (social, religious, and legal) reasons was not significantly different by knowledge level (*p* = .057). Moreover, perceptions on SC use, prevalence and ease of access were significantly different among groups by gender (*p* = .028), smoking (p = .024), being informed by social media (p = .002), and by the level of knowledge of SC (*p* = .001).

## Discussion

In this study, and to the author’s best knowledge, the level of knowledge along with the perceptions of undergraduate students regarding SC were reported for the first time in Jordan and the Middle East.

Students showed an excellent level of self-reported familiarity but with low levels of knowledge of SC in contrast to previous reports from other countries [18, 20, 21]. This could be due to the considerably large media coverage and official governmental discourse in Jordan during the last couple of years. Misconceptions were mostly focused on the composition of the SC and their effects, consistent with a previous study with similar findings [22]. These misconceptions may be attributed to the local media coverage of the SC crisis and outbreak of SC-related crimes in Jordan between 2014–2016. Similarly, the role of the media may explain why there were no differences in level of knowledge and predictors of good knowledge by gender, as the impact of the SC use outbreak and SC-related crimes revibrated through the whole Jordanian society.

Data analysis showed that participants who were informed by both traditional and social media platforms of SC use and effects were more likely to have good knowledge of SC. The findings by Beyhun and colleagues who reported that social media had a positive effect in enhancing the awareness regarding SC were similar to those results in the current study [18]. The use of social media was useful as a potential intervention in smoking cessation programs [23].

In the current study, knowledge of SC is associated with substance use such as smoking and alcohol. Data from the World Health Organization (WHO) suggested that at least 1 in every 3 males in Jordan were daily smokers [24] where Jordan was the leading country in smoking prevalence in the region. This is also similarly seen among university students [25]. Nevertheless, individuals who were engaged in substance abuse and polydrug were more likely to be interested in other substance abuse [17]. This is congruent with evidence suggesting that SC use was associated with those who were users of other substances and that the substance use progresses from licit drugs to other illicit drugs in what is known as gateway effect [17, 26, 27].

Generally, the adverse effects and health consequences of taking SC are widely known to the students. It is evident that students were aware of the re-emergence of SC in various forms in the local market and the issues related to its laboratory identification. Due to the constant changing of SC products in terms of their chemical blends and the inclusion of some structures that are poorly characterized, detecting SC stands as a challenging issue. It has been reported that this difficulty in detecting SC is a factor behind why many young users opt for SC use in an attempt to circumvent legal consequences [17, 22]. In Jordan, the 2016 Drug and Psychoactive Substances Act was enacted to circumscribe this barrier by even going to the lengths of banning any precursors that may be used to manufacture new SC products, which was seen as a substantial progress in a series of regulations that are being put to combat SC use in Jordan [14].

Research has recognized that curiosity to try new substances is one of the factors that increase SC use [17]. Nevertheless, the current study described the perceived justifications against the use of SC along with the perceived SC use prevalence and ease of access. Several aspects evince in defining the students’ ethical perceptions against the use of SC where it emerges as a multifaceted concept. Most students agreed that their use is socially, ethically, and legally rejected. In the current study, there were significant differences in the ethical perceptions against the use of SC detected by gender, smoking or alcohol use and being informed by the media, but there were no differences by the level of knowledge. Those of good knowledge and those of low knowledge did not perceive using SC to be ethically compromisable. This is consistent with a nationally representative survey that was performed on high school students in the United States [28]. Another study conducted on 10,528 college students in the United States, where the effect of religion as evaluated by religious observance through attendance of regular services, showed that those who attended less than twice a month were more likely to attempt using SC [29]. In Jordan, the national strategy against the use of drugs (2009) adopted several ways of ‘self-immunization’ among which is instilling religious, traditional, and social values as means of spreading awareness against the use of psychoactive drugs [30]. Noticeably, it has been reported that many SC users perceived them to be legal alternatives to other illicit drugs; the findings of this study seem not to support this. This may stem from the role of local media campaigns to raise awareness amid the 2016 SC crisis.

The main strength of this study was the large sample of participants, who were representative of young Jordanians, surveyed at a ratio of participants to items equal to 99:1. Therefore, advanced statistical methods were applied with enough power to support the main findings. However, our findings were limited. At first, it was not possible to estimate the prevalence of SC use among university students in Jordan due to several reasons including poor reporting from local hospitals and authorities and confidentiality of users. Future research could estimate the national prevalence and trends regarding SC use in Jordon.

Secondly, while this study identified that media had a positive role in raising awareness of the dangers of SC use, it also prompted the question about the role of spreading information regarding SC during the SC outbreak in 2014–2016. For instance, the results of the study suggested high familiarity with SC but also a low level of knowledge. Future research could explore at length the mediating role of media, if any, or any other factor in spreading information regarding SC in Jordan.

## Conclusions

This study showed that there was a low level of knowledge regarding SC among university students in Jordan, which may play a role in the use of SC in the country. Herein, many opportunities exist for public health education to raise awareness against SC use. Moreover, students perceived SC use to be prevalent and ethically unacceptable, for religious, social, and legal reasons. This research will be instrumental in developing policies for SC use prevention and hazard reduction in Jordan and other Middle Eastern countries.

## Supporting information

S1 Table The final version of the questionnaire in Arabic. (DOCX)

S2 Table The final version of the questionnaire in English. (DOCX)

S3 Table Rotated component matrix and factorial structure of students’ perceptions towards SC. (DOCX)

S4 Table Unadjusted odds ratios and 95% confidence interval for predictors of good knowledge on synthetic cannabinoids from univariate analysis. (DOCX)

## References

[1] Castaneto MS, Gorelick DA, Desrosiers NA, Hartman RL, Pirard S, Huestis MA. Synthetic cannabinoids: Epidemiology, pharmacodynamics, and clinical implications. Drug Alcohol Depend. 2014;144:12–41. doi: 10.1016/j.drugalcdep.2014.08.005 ; PubMed Central PMCID: PMC4253059. 25220897 PMC4253059

[2] Wiley JL, Marusich JA, Martin BR, Huffman JW. 1-Pentyl-3-phenylacetylindoles and JWH-018 share in vivo cannabinoid profiles in mice. Drug Alcohol Depend. 2012;123(1):148–153. doi: 10.1016/j.drugalcdep.2011.11.001 ; PubMed Central PMCID: PMC3294131. 22127210 PMC3294131

[3] Wiley JL, Marusich JA, Huffman JW. Moving around the molecule: Relationship between chemical structure and in vivo activity of synthetic cannabinoids. Life Sci. 2014;97(1):55–63. doi: 10.1016/j.lfs.2013.09.011 ; PubMed Central PMCID: PMC3944940. 24071522 PMC3944940

[4] Spaderna M, Addy PH, D’Souza DC. Spicing things up: synthetic cannabinoids. Psychopharmacology (Berl). 2013;228(4):525–540. doi: 10.1007/s00213-013-3188-4 ; PubMed Central PMCID: PMC3799955. 23836028 PMC3799955

[5] Huffman JW. Cannabimimetic indoles, pyrroles, and indenes: Structure–activity relationships and receptor interactions. In: Reggio PH, editor. The Cannabinoid Receptors. Totowa, NJ: Humana Press; 2009. pp. 49–94.

[6] United Nations Office on Drugs and Crime. Synthetic Cannabinoids in Herbal Products 2011 [Cited 2019 May 26]. Available from: https://www.unodc.org/documents/scientific/Synthetic_Cannabinoids.pdf.

[7] Cooks T, Davis TK, Hu J, Metheny R, Schwartz M, Gerona R. “Smoking” guns: Questions. Pediatr Nephrol. 2016;31(1):61–62. doi: 10.1007/s00467-014-2978-1 . 25330878

[8] Auwärter V, Dresen S, Weinmann W, Müller M, Pütz M, Ferreirós N. ‘Spice’ and other herbal blends: Harmless incense or cannabinoid designer drugs? J Mass Spectrom. 2009;44(5):832–837. doi: 10.1002/jms.1558 . 19189348

[9] Uchiyama N, Kikura-Hanajiri R, Kawahara N, Haishima Y, Goda Y. Identification of a cannabinoid analog as a new type of designer drug in a herbal product. Chem Pharm Bull. 2009;57(4):439–441. doi: 10.1248/cpb.57.439 . 19336947

[10] Brents LK, Prather PL. The K2/Spice Phenomenon: emergence, identification, legislation and metabolic characterization of synthetic cannabinoids in herbal incense products. Drug Metab Rev. 2014;46(1):72–85. doi: 10.3109/03602532.2013.839700 ; PubMed Central PMCID: PMC4100246. 24063277 PMC4100246

[11] U.S. Department of Justice, Drug Enforcement Administration: National Forensic Laboratory Information System. Synthetic Cannabinoids and Synthetic Cathinones Reported in NFLIS, 2010–2013 Springfield, VA, USA: U.S. Department of Justice; 2014 [Cited 2019 Sepetmber 8]. Available from: https://www.nflis.deadiversion.usdoj.gov/desktopmodules/reportdownloads/reports/syncannabsyncath.pdf.

[12] U.S. Department of Justice, Drug Enforcement Administration: National Forensic Laboratory Information System. Synthetic Cannabinoids and Synthetic Cathinones Reported in NFLIS, 2013–2015 Springfield, VA, USA: U.S. Department of Justice; 2016 [Cited 2019 October 12]. Available from: https://www.nflis.deadiversion.usdoj.gov/DesktopModules/ReportDownloads/Reports/NFLIS-SR-SynthCannabinoidCathinone.pdf.

[13] Forensic and Criminal Evidence Lab. Public Security Directorate; 2016 [Cited 2017 January 8]. Available from: https://www.psd.gov.jo/index.php/ar/2015-03-17-08-29-01.

[14] Ajunaidi S. Legal Review on “Drugs and Psychotropic Substances Act Number (23) for the Year 2016 [Cited 2019 Oct 12]. Available from: http://www.nchr.org.jo/User_Site/Site/View_ArticleAr.aspx?type=2&ID=1522.

[15] Bybaris S. An Official Study Reveals Exciting Information about Drugs in Jordan. Al Ghad. 2016 Nov 16 [Cited 2019 September 3]. Available from: https://alghad.com/%D8%AF%D8%B1%D8%A7%D8%B3%D8%A9-%D8%B1%D8%B3%D9%85%D9%8A%D8%A9-%D8%AA%D9%83%D8%B4%D9%81-%D9%85%D8%B9%D9%84%D9%88%D9%85%D8%A7%D8%AA-%D9%85%D8%AB%D9%8A%D8%B1%D8%A9-%D8%B9%D9%86-%D8%A7%D9%84%D9%85%D8%AE/.

[16] Drug Rehabilitation Center, Drug Enforcement Administration. Public Security Directorate; [Cited 2017 April 12]. Available from: https://www.psd.gov.jo/index.php/ar/2015-03-02-09-22-55.

[17] Loeffler G, Delaney E, Hann M. International trends in spice use: Prevalence, motivation for use, relationship to other substances, and perception of use and safety for synthetic cannabinoids. Brain Res Bull. 2016;126:8–28. doi: 10.1016/j.brainresbull.2016.04.013 . 27108542

[18] Beyhun NE, Can G, Topbas M, Cankaya S, Ketenci HC. Are the last grade medical students aware of the danger of synthetic cannabinoids? J Forensic Leg Med. 2016;38:1–5. doi: 10.1016/j.jflm.2015.11.014 . 26694870

[19] Sullivan GM, Artino AR Jr. Analyzing and interpreting data from likert-type scales. J Grad Med Educ. 2013;5(4):541–542. Epub 2014/01/24. doi: 10.4300/JGME-5-4-18 ; PubMed Central PMCID: PMC3886444. 24454995 PMC3886444

[20] Stephens JL. Synthetic cannabinoid usage among college students: The example of K2 and spice [Master of Science]. Denton, TX, USA: University of North Texas; 2011. Available from: https://digital.library.unt.edu/ark:/67531/metadc84283/

[21] Heckman CJ, Dykstra JL, Collins BN. Substance-related knowledge, attitude, and behaviour among college students: Opportunities for health education. Health Educ J. 2011;70(4):383–399. doi: 10.1177/0017896910379694 ; PubMed Central PMCID: PMC3268229. 22303033 PMC3268229

[22] Salani DA, Zdanowicz MM. Synthetic cannabinoids: The dangers of spicing it up. J Psychosoc Nurs Ment Health Serv. 2015;53(5):36–43. Epub 2015/05/15. doi: 10.3928/02793695-20150422-01 . 25974923

[23] Pechmann C, Pan L, Delucchi K, Lakon CM, Prochaska JJ. Development of a twitter-based intervention for smoking cessation that encourages high-quality social media interactions via automessages. J Med Internet Res. 2015;17(2):e50. doi: 10.2196/jmir.3772 ; PubMed Central PMCID: PMC4376170. 25707037 PMC4376170

[24] World Health Organization. WHO Report on the Global Tobacco Epidemic, 2017: World Health Organization; 2017 [Cited 2019 September 3]. Available from: https://www.who.int/tobacco/surveillance/policy/country_profile/jor.pdf.

[25] Khader YS, Alsadi AA. Smoking habits among university students in Jordan: Prevalence and associated factors. East Mediterr Health J. 2008;14(4):897–904. . 19166173

[26] Baggio S, Studer J, Mohler-Kuo M, Daeppen JB, Gmel G. Profiles of drug users in Switzerland and effects of early-onset intensive use of alcohol, tobacco and cannabis on other illicit drug use. Swiss Med Wkly. 2013;143:w13805. Epub 2013/06/07. doi: 10.4414/smw.2013.13805 . 23740102

[27] Kandel D, Kandel E. The Gateway hypothesis of substance abuse: developmental, biological and societal perspectives. Acta Paediatr. 2015;104(2):130–137. doi: 10.1111/apa.12851 . 25377988

[28] Palamar JJ, Acosta P. Synthetic cannabinoid use in a nationally representative sample of US high school seniors. Drug Alcohol Depend. 2015;149:194–202. doi: 10.1016/j.drugalcdep.2015.01.044 ; PubMed Central PMCID: PMC4361370. 25736618 PMC4361370

[29] Egan KL, Suerken CK, Reboussin BA, Spangler J, Wagoner KG, Sutfin EL, et al. K2 and Spice use among a cohort of college students in southeast region of the USA. Am J Drug Alcohol Abuse. 2015;41(4):317–322. doi: 10.3109/00952990.2015.1043438 ; PubMed Central PMCID: PMC4526379. 26030768 PMC4526379

[30] Rochefoucauld MdL. Jordan: Drug Situation and Policy: Council of Europe; 2014 [Cited 2019 November 15]. Available from: https://rm.coe.int/drug-situation-and-policy-by-matthieu-de-la-rochefoucauld/168075f2a7.

