# Supplementary material for: Knowledge and perceptions of synthetic cannabinoids among university students in Jordan
Source: PLoS One. 2021 Jun 24;16(6):e0253632. doi: 10.1371/journal.pone.0253632 (PMC8224919; doi:10.1371/journal.pone.0253632)
Supplement: S1 Table — (DOCX) [file pone.0253632.s001.docx]

| **S1 Table. The Final Version of the Questionnaire in Arabic.** | | | | | | | | | | |
| --- | --- | --- | --- | --- | --- | --- | --- | --- | --- | --- |
| نتقدم من جميع المشاركين معنا في هذا البحث بجزيل الشكر وعظيم الامتنان. ونود أن نبين ما يلي:   - الهدف من هذا الاستبيان هو دراسة معرفة وتصورات طلبة الجامعات الأردنية حول القنيبات المصنعة (الجوكر). - يشرف على هذا الدراسة العلمية الأكاديمية مجموعة من الباحثين من كلية الصيدلة في جامعة العلوم والتكنولوجيا الأردنية. - المشاركة في هذا الدراسة تطوعية. - مشاركتك في هذه الدراسة ستبقى سرية، ولا يوجد ما يكشف عن هويتك. - يستغرق تعبئة الاستبيان نحو نحو 10 دقائق، ولك حرية تجاوز الأسئلة التي لا ترغب بالإجابة عليها، كما ويمكنك التوقف عن المشاركة في هذه الدراسة في أي وقت. | | | | | | | | | | |
| **المعلومات الديموغرافية** | | | | | | | | | | |
| الجنس | | | 🞎 ذكر 🞎 أنثى | | | | | | | |
| العمر | | | 🞎 أقل من 20 عام 🞎 22-23 عام 🞎 24 عام فأكثر | | | | | | | |
| اسم الجامعة | | | 🞎 اليرموك 🞎 العلوم التطبيقية الخاصة 🞎 الهاشمية 🞎 البوليتكنك 🞎 الزرقاء الخاصة  🞎 البترا 🞎 الأردنية 🞎 العلوم والتكنولوجيا 🞎 مؤتة 🞎 آل البيت | | | | | | | |
| الديانة | | | 🞎 الإسلام 🞎 المسيحية 🞎 غير ذلك | | | | | | | |
| التخصص | | | 🞎 طبي 🞎 هندسي 🞎 شرعي/قانوني 🞎 تقني/حاسوب  🞎 إنساني/تربوي 🞎 اقتصاد/إعلام 🞎 زراعي 🞎 علوم وآداب  🞎 غير ذلك، اذكره ................................................................. | | | | | | | |
| السنة الدراسية | | | 🞎 الأولى 🞎 الثانية 🞎 الثالثة 🞎 الرابعة 🞎 الخامسة 🞎 السادسة | | | | | | | |
| مكان الإقامة | | | 🞎 عمان 🞎 إربد 🞎 المفرق 🞎 البلقاء 🞎 مأدبا 🞎 الزرقاء  🞎 جرش 🞎 عجلون 🞎 الكرك 🞎 معان 🞎 الطفيلة 🞎 العقبة | | | | | | | |
| 1. هل أنت مدخن للسجائر؟ | | | | 🞎 لا 🞎 نعم 🞎 مدخن سابق | | | | | | |
| 1. هل تتناول الأرجيلة؟ | | | | 🞎 لا 🞎 نعم 🞎 سابقاً | | | | | | |
| 1. هل سمعت مسبقاً عن الجوكر (القنيبات الصناعية)؟ | | | | 🞎 لا 🞎 نعم | | | | | | |
| 1. اذا سبق وسمعت عن الجوكر، ما هي مصادر معلوماتك عنه؟ | | | | 🞎 وسائل الإعلام المختلفة كالتلفزيون، والراديو، والجرائد  🞎 مواقع التواصل الاجتماعي: Twitter، Facebook، WhatsApp  🞎 من الناس كالعائلة، والأصدقاء، والجيران  🞎 من مختصين بالرعاية الصحية كالأطباء والصيادلة والممرضين  🞎 محاضرات توعوية وندوات | | | | | | |
| 1. هل تعرف أي شخص يتناول الجوكر؟ | | | | 🞎 لا 🞎 نعم | | | | | | |
| 1. هل تتناول الكحول؟ | | | | 🞎 لا 🞎 نعم | | | | | | |
| **المعرفة حول القنيبات الاصطناعية (الجوكر)**  ضع إشارة صح بجانب كل جملة تراها صحيحة، وإشارة خطأ أمام كل جملة تراها خاطئة حول القنيبات الاصطناعية (الجوكر). | | | | | | | | | | |
| **الرقم** | | **الجملة** | | | | **صحيح** | | **غير صحيح** | | |
|  | | الجوكر عبارة عن منتجات نباتية | | | |  | |  | | |
|  | | الجوكر عبارة عن ادوية علاجية | | | |  | |  | | |
|  | | يتم استخدام المبيدات الحشرية في تركيب الجوكر | | | |  | |  | | |
|  | | يعتبر الجوكر أكثر فعالية من الحشيش | | | |  | |  | | |
|  | | يعتبر الجوكر رخيص الثمن | | | |  | |  | | |
|  | | يسبب تعاطي الجوكر الموت | | | |  | |  | | |
|  | | يحدث تعاطي الجوكر تغييرات سلوكية | | | |  | |  | | |
|  | | يسبب تعاطي الجوكر مشاكل صحية مختلفة | | | |  | |  | | |
|  | | من الصعب الكشف عن تعاطي الجوكر من خلال اختبارات الدم أو البول | | | |  | |  | | |
|  | | تتغير منتجات الجوكر التي تطرح في السوق بشكل مستمر | | | |  | |  | | |
| **التصورات حول القنيبات الاصطناعية (الجوكر)** | | | | | | | | | | |
|  |  | | | | **موافق بشدة** | **موافق** | **محايد** | | **لا اوافق** | **لا اوافق بشدة** |
|  | من السهل الحصول على الجوكر في السوق المحلي | | | |  |  |  | |  |  |
|  | يعتبر تعاطي الجوكر سلوك محرّم دينياً | | | |  |  |  | |  |  |
|  | يعتبر تعاطي الجوكر سلوك مرفوض اجتماعياً | | | |  |  |  | |  |  |
|  | يعتبر تعاطي الجوكر سلوك ممنوع بالقانون | | | |  |  |  | |  |  |
|  | يعتبر تعاطي الجوكر حرية شخصية | | | |  |  |  | |  |  |
|  | يمكن استخدام وسائل التواصل الاجتماعي للتوعية من مخاطر استخدام الجوكر | | | |  |  |  | |  |  |
|  | تزيد التقارير المتعلقة بمنتجات الجوكر في وسائط الإعلام والتلفزيون من الفضول في محاولة تجريب الجوكر | | | |  |  |  | |  |  |
|  | ينتشر استخدام الجوكر بين طلاب الجامعات بكثرة | | | |  |  |  | |  |  |
