# Supplementary material for: Knowledge and perceptions of synthetic cannabinoids among university students in Jordan
Source: PLoS One. 2021 Jun 24;16(6):e0253632. doi: 10.1371/journal.pone.0253632 (PMC8224919; doi:10.1371/journal.pone.0253632)
Supplement: S2 Table — (DOCX) [file pone.0253632.s002.docx]

| **S2 Table. The Final Version of the Questionnaire in English.** | | | | | | | | | |
| --- | --- | --- | --- | --- | --- | --- | --- | --- | --- |
| We are pleased to thank all the participants in this research, and would like to emphasize the following:   - The aim of this study is to investigate the knowledge and perceptions of university students on Synthetic Cannabinoids (JOKER) - This study is supervised by a group of academic researchers from the Faculty of Pharmacy at the Jordan University of Science and Technology - Participation in this study is voluntary. - Your participation in this study shall remain confidential, and none of the data can reveal your identity. - This questionnaire requires up to 10 minutes to complete, and you are completely free to not to respond to any of question items, and you can withdraw/ stop participating in this study at any time. | | | | | | | | | |
| **Demographic information** | | | | | | | | | |
| Gender | 🞎 Male 🞎 Female | | | | | | | | |
| Age group | 🞎 <20 years 🞎 22-23 years 🞎 >24 years | | | | | | | | |
| University Name | 🞎 Yarmouk University 🞎 Applied Science University  🞎 Hashemite University 🞎 Polytechnic University  🞎 Zarqa’ University 🞎 Al-Bayt University  🞎 Mo’uta University 🞎 Jordan University of Science and Technology  🞎 Petra University 🞎 University of Jordan | | | | | | | | |
| Religion | 🞎 Islam 🞎 Christianity 🞎 other | | | | | | | | |
| Specialty | 🞎 Medical /health 🞎 Engineering  🞎 legal studies 🞎 Computer Science  🞎 Humanities and education 🞎 Economy and media 🞎 Agriculture 🞎 Arts and sciences 🞎 other: …………. | | | | | | | | |
| Year of Study | 🞎 1^st^ year 🞎 2^nd^ year 🞎 3^rd^ year 🞎4^th^ year 🞎5^th^ year 🞎 6^th^ year | | | | | | | | |
| Residency | 🞎 Amman 🞎 Irbid 🞎 Mafraq 🞎 Balqa’ 🞎 Madaba 🞎 Az-zarqa’  🞎 Jarash 🞎 Ajloun 🞎 Karak 🞎 Ma’an 🞎 Tafilah 🞎 Aqaba | | | | | | | | |
| Do you smoke tobacco? Choose yes if you are a lifetime smoker. | | | 🞎 Yes (currently) 🞎 No 🞎 Ex-smoker | | | | | | |
| Do you consume waterpipe? | | | 🞎 Yes (currently) 🞎 No 🞎 Ex-consumer | | | | | | |
| Have you heard before about Synthetic Cannabinoids (JOKER)? | | | 🞎 Yes 🞎 No | | | | | | |
| If you have ever heard about Synthetic Cannabinoids (JOKER), were you informed by any of these? | | | 🞎 traditional media (TV/ Radio/ Newspapers)  🞎 Social media: Twitter, Facebook, WhatsApp  🞎 From people: family, friends, and neighbors  🞎 From healthcare providers (physicians, pharmacists, and nurses)  🞎 educational and awareness lectures | | | | | | |
| Do you know someone who consumes Synthetic Cannabinoids (JOKER)? | | | 🞎 Yes 🞎 No | | | | | | |
| Do you consume alcohol? | | | 🞎 Yes 🞎 No | | | | | | |
| **Knowledge on Synthetic Cannabinoids (JOKER):** To each of the following, indicate whether you think the statement is true or false. | | | | | | | | | |
| **Statement** | | | | | | **True** | | **False** | |
| 1. Synthetic cannabinoids are herbal substances | | | | | |  | |  | |
| 1. Synthetic cannabinoids are drugs of pharmacological effect | | | | | |  | |  | |
| 1. Insecticides are used in manufacturing synthetic cannabinoids products | | | | | |  | |  | |
| 1. Synthetic cannabinoids are more potent than cannabis/ hashish | | | | | |  | |  | |
| 1. Synthetic cannabinoids products are cheap | | | | | |  | |  | |
| 1. Synthetic cannabinoids may cause death | | | | | |  | |  | |
| 1. Synthetic cannabinoids may cause behavioral changes | | | | | |  | |  | |
| 1. Synthetic cannabinoids may cause health problems | | | | | |  | |  | |
| 1. Synthetic cannabinoids are hard to detect in blood or urine tests | | | | | |  | |  | |
| 1. Marketed products containing synthetic cannabinoids are constantly changing | | | | | |  | |  | |
| **Perceptions of Synthetic Cannabinoids (JOKER)** | | | | | | | | | |
| **Statement** | | **Strongly Agree** | | **Agree** | **Neutral** | | **Disagree** | | **Strongly disagree** |
| 1. Taking synthetic cannabinoids is considered a behavior banned by religion | |  | |  |  | |  | |  |
| 1. Taking synthetic cannabinoids is considered a behavior rejected by social norms | |  | |  |  | |  | |  |
| 1. Taking synthetic cannabinoids is considered a behavior banned by law | |  | |  |  | |  | |  |
| 1. Taking synthetic cannabinoids is considered a freedom of choice for the individual | |  | |  |  | |  | |  |
| 1. It is easy to obtain synthetic cannabinoids from the local market | |  | |  |  | |  | |  |
| 1. The media reports on synthetic cannabinoids increase people’s curiosity to attempt taking synthetic cannabinoids | |  | |  |  | |  | |  |
| 1. The act of taking synthetic cannabinoids is widely spread among university students | |  | |  |  | |  | |  |
| 1. Social media can be utilized to spread awareness on the risks of taking synthetic cannabinoids | |  | |  |  | |  | |  |
