# Supplementary material for: Knowledge and perceptions of synthetic cannabinoids among university students in Jordan
Source: PLoS One. 2021 Jun 24;16(6):e0253632. doi: 10.1371/journal.pone.0253632 (PMC8224919; doi:10.1371/journal.pone.0253632)
Supplement: S3 Table — (DOCX) [file pone.0253632.s003.docx]

| **S3 Table. Rotated Component Matrix and Factorial Structure of Students’ Perceptions Towards SC.** |
| --- |

| **Statement** | ***Factor 1*** | ***Factor 2*** |
| --- | --- | --- |
| It is easy to obtain SC from the local market |  | 0.728 |
| Taking SC is considered a behaviour banned by religion | 0.647 |  |
| Taking SC is considered a behaviour rejected by social norms | 0.830 |  |
| Taking SC is considered a behaviour banned by law | 0.752 |  |
| Taking SC is considered a freedom of choice for the individual | -0.534 |  |
| Social media can be utilized to spread awareness on the risks of taking SC |  | 0.638 |
| The media reports on SC increase people’s curiosity to attempt taking SC |  | 0.583 |
| The act of taking SC is widely spread among university students |  | 0.736 |
|  | **46.9** | **19.4** |
|  | **46.9** | **66.3** |

Bartlett’s test of sphericity was significant (*X*^2^ (28) = 1999.4, *p*<.0001) and Kaiser-Meyer-Olkin measure of sampling adequacy was 0.692, which renders the data eligible for factorability. All items had communalities greater than 0.6. The solution was confirmed by parallel analysis conducted in JASP statistical package.
