# Supplementary material for: Knowledge and perceptions of synthetic cannabinoids among university students in Jordan
Source: PLoS One. 2021 Jun 24;16(6):e0253632. doi: 10.1371/journal.pone.0253632 (PMC8224919; doi:10.1371/journal.pone.0253632)
Supplement: S4 Table — (DOCX) [file pone.0253632.s004.docx]

| **S4 Table: Unadjusted Odds Ratios and 95% Confidence Interval for Predictors of Good Knowledge on Synthetic Cannabinoids** | | |
| --- | --- | --- |
| **Variable** | **Univariate analysis** | |
|  | **Unadjusted OR (95% CI)** | ***p*-value** |
| **Gender** |  |  |
| Male | Referent |  |
| Female | 0.640 (0.536-0.763) | <.0001 |
| **Age** |  |  |
| <20 years | Referent |  |
| 20-23 years | 0.829 (0.579-1.186) | .304 |
| >24 years | 1.035 (0.738-1.453) | .804 |
| **Smoking/ Waterpipe (life-time)** |  |  |
| Non-smoker | Referent |  |
| Smoker | 1.249 (1.044-1.495) | .015 |
| Ex-smoker | 1.207 (0.843-1.728) | .304 |
| **Self-reported Familiarity with SC** |  |  |
| No | Referent |  |
| Yes | 3.002 (1.996-4.515) | .0001 |
| **Alcohol consumption** |  |  |
| No | Referent |  |
| Yes | 2.703 (1.844-3.962) | <.0001 |
| **Informed by traditional media** |  |  |
| No | Referent |  |
| Yes | 1.473 (1.256-1.726) | <.0001 |
| **Informed by social media** |  |  |
| No | Referent |  |
| Yes | 1.332 (1.135-1.562) | <.0001 |
| **Field of study** |  |  |
| Health | Referent |  |
| Engineering | 1.043 (0.839-1.296) | .707 |
| Law, Humanities and Educational Sciences | 0.552 (0.424-0.720) | <.0001 |
| Technology | 0.749 (0.535-1.050) | .093 |
| Administrative | 0.889 (0.674-1.174) | .407 |
| Arts and others | 0.694 (0.545-0.884) | .003 |
| **Residency** |  |  |
| Northern Jordan | Referent |  |
| Central Jordan | 1.388 (1.145-1.682) | .001 |
| Southern Jordan | 1.213 (0.964-1.527) | .100 |
